# Association of vitamin D levels with metabolic dysfunction-associated fatty liver disease in children aged 12–18 years

**DOI:** 10.3389/fnut.2025.1615851

**Published:** 2025-11-04

**Authors:** Xuejie Gao, Yuyun Chen, Xinrui Wang, Yuehang Chen, Xiaoyan Chen, Haibo Li, Hong Ye

**Affiliations:** ^1^Department of Digestive & Nutrition, Fujian Children's Hospital (Fujian Branch of Shanghai Children's Medical Center), College of Clinical Medicine for Obstetrics & Gynecology and Pediatrics, Fujian Medical University, Fuzhou, Fujian, China; ^2^Fujian Maternity and Child Health Hospital, College of Clinical Medicine for Obstetrics & Gynecology and Pediatrics, Fujian Medical University, Fuzhou, Fujian, China

**Keywords:** MAFLD, vitamin D, adolescents, NHANES, retinol status

## Abstract

**Objective:**

This study examines the association between serum vitamin D levels and the prevalence of metabolic dysfunction-associated fatty liver disease (MAFLD) in adolescents, along with potential modifying factors.

**Methods:**

Data from 950 adolescents aged 12–18 years in the National Health and Nutrition Examination Survey (NHANES) 2017–2018 were analyzed. MAFLD was defined using hepatic steatosis and metabolic dysfunction criteria. Serum 25(OH)D levels were measured, and weighted logistic regression and restricted cubic spline models were applied to assess their association with MAFLD risk. Stratified analyses were also conducted.

**Results:**

Lower serum 25(OH)D levels were significantly associated with higher MAFLD risk (p < 0.001), showing a nonlinear dose-response relationship. Adolescents with 25(OH)D ≥ 75 nmol/L had a 57% lower risk of MAFLD compared to those with levels < 50 nmol/L. Stratified analysis indicated that the protective effect of vitamin D was more evident in individuals with higher retinol levels, though retinol alone was not significantly associated with MAFLD.

**Conclusion:**

Vitamin D deficiency is significantly associated with MAFLD in adolescents, with a nonlinear dose-response relationship modulated by retinol status. These findings underscore the potential role of vitamin D in MAFLD prevention and provide a basis for further prospective or intervention studies.

## Introduction

Metabolic dysfunction-associated fatty liver disease (MAFLD), formerly named non-alcoholic fatty liver disease (NAFLD), is a significant chronic liver disease worldwide. A recent meta-analysis revealed that the global prevalence of MAFLD is 30%, reflecting a 50% increase from 1990 to 2019 (1). This increase presents a notable public health burden (2). Notably, with the socioeconomic transformation and lifestyle changes, the prevalence of MAFLD in adolescents has shown a significant trend of younger age (3, 4). The primary pathological feature of MAFLD is fat deposition in hepatocytes (≥5%), driven by metabolic disorders (5). This pathological process is closely linked to abnormal liver function and significantly raises the risk of developing type 2 diabetes and cardiovascular diseases in adulthood through mechanisms such as insulin resistance and chronic inflammation (6). Therefore, MAFLD poses a long-term threat to public health.

As a fat-soluble molecule with steroid hormone characteristics (7), the multi-dimensional metabolic regulatory functions of vitamin D are gradually being uncovered, revealing its extensive physiological roles. Previous studies have established the central role of vitamin D in bone mineral metabolism, maintaining bone health by promoting intestinal calcium absorption and regulating bone remodeling (8, 9). Recent research has shown that vitamin D is broadly involved in physiological processes such as immune regulation, hormone secretion, and cell proliferation and differentiation (10). Vitamin D receptors are widely distributed in the liver, adipose tissue, and immune cells, where they play a crucial role in maintaining metabolic homeostasis by regulating glucose and lipid metabolism, suppressing the release of proinflammatory factors, and improving insulin sensitivity (11). In the liver, vitamin D is metabolized by 25-hydroxylase (CYP2R1) into 25-hydroxyvitamin D [25(OH)D] (12). Vitamin D status is typically classified according to serum 25(OH)D concentrations. Generally, vitamin D deficiency is defined as serum 25(OH)D levels below 20 ng/mL (50 nmol/L), while insufficiency refers to serum concentrations between 20 and 30 ng/mL (50–75 nmol/L). By contrast, vitamin D sufficiency is generally considered to be achieved when serum 25(OH)D levels exceed 30 ng/mL (75 nmol/L) (13).

Several studies have suggested that vitamin D deficiency may play a critical role in the development and progression of various metabolic diseases, including insulin resistance, obesity, hypertension, metabolic syndrome, and cardiovascular disease (14–17). In addition, the association between vitamin D levels and NAFLD has attracted increasing attention (18). A cross-sectional study revealed that low serum 25(OH)D levels in the general population are associated with an increased risk of NAFLD (19). Furthermore, a study from Korea indicated that vitamin D deficiency may be considered an independent risk factor for suspected NAFLD in adolescents (20). Besides, obesity constitutes one of the primary risk factors for MAFLD. It significantly increases the risk of developing MAFLD by promoting hepatic fat accumulation and contributing to metabolic dysfunction (21). Concurrently, obesity is also associated with vitamin D deficiency (22), suggesting that vitamin D may mediate the effect of obesity on MAFLD pathogenesis. However, research on the association between MAFLD and vitamin D in adolescents aged 12–18 years remains relatively limited.

Based on the current research advancements in the potential role of vitamin D in metabolic diseases, this study proposes the scientific hypothesis that adolescents with lower 25(OH)D levels may be more susceptible to MAFLD. Given the new nomenclature and diagnostic criteria for MAFLD, the population with MAFLD differs from that with NAFLD in many respects (23). Therefore, it is necessary to further clarify the characteristics and influencing factors of MAFLD. This study utilizes data from the 2017–2018 National Health and Nutrition Examination Survey (NHANES) to investigate the association between serum 25(OH)D status and MAFLD, and to further explore potential effect modifiers.

## Material and methods

### Study population

**Figure 1:**
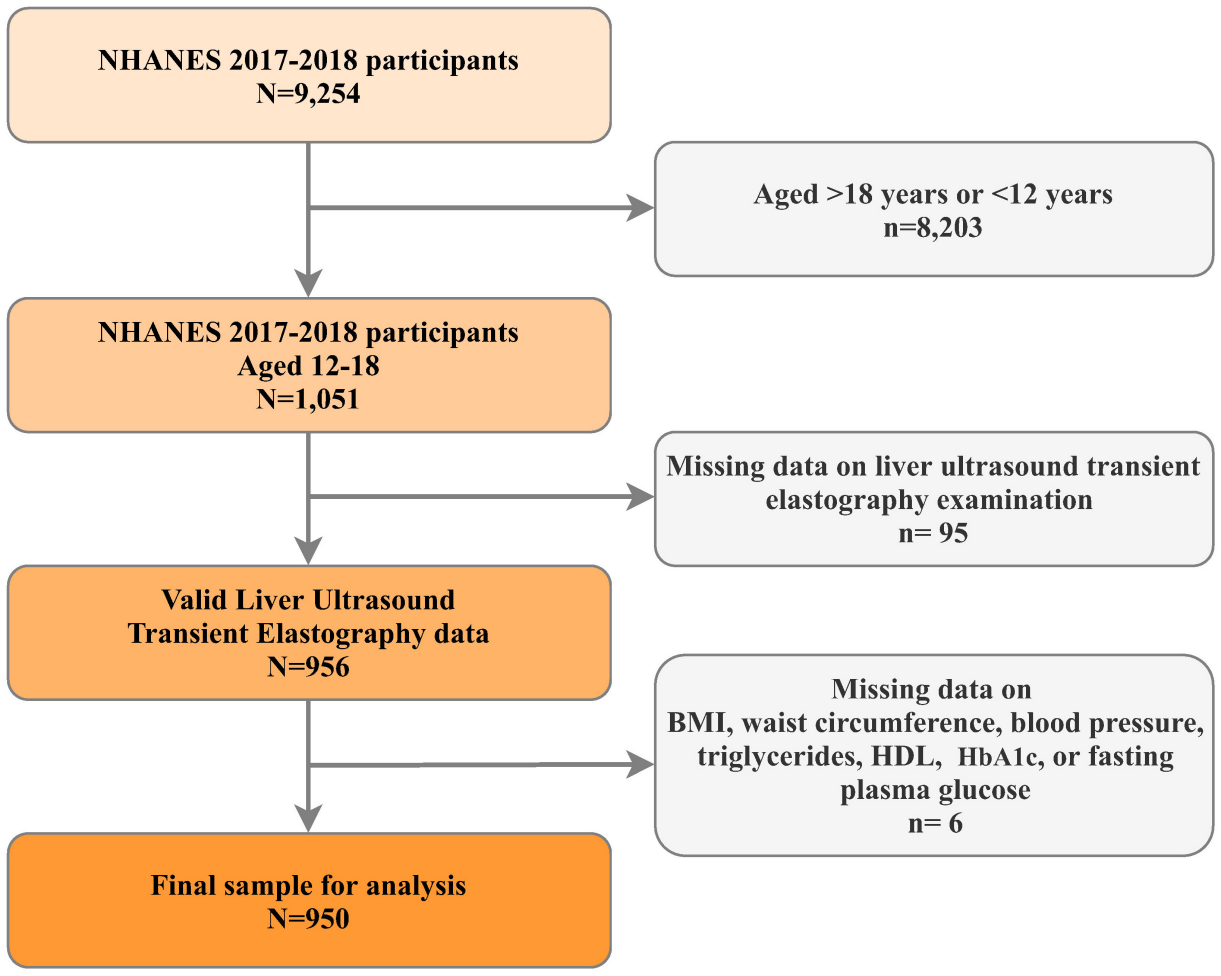
Flowchart of participant inclusion and exclusion.

The National Health and Nutrition Examination Survey (NHANES) represents a continuous research initiative designed to evaluate health and dietary conditions across a diverse, non-institutionalized U.S. population through its sophisticated, multistage probability sampling framework (24). For this investigation, we utilized data from the 2017–2018 NHANES cycle, specifically chosen for its inclusion of liver ultrasound transient electrography assessments. Our analysis focused on adolescents aged 12–18 years. Participants lacking essential data points were excluded, including those without liver ultrasound transient electrography results and missing critical metabolic dysfunction indicators such as body mass index (BMI), waist circumference (WC), high-density lipoprotein (HDL) -cholesterol levels, blood pressure (BP) readings (both systolic and diastolic), hemoglobin A1C (HbA1C), and fasting plasma glucose (FPG) measurements (Figure 1). The National Center for Health Statistics (NCHS) oversees the administration and maintenance of NHANES. The NCHS Institutional Review Board/Ethic Review Board granted approval for the NHANES protocols (Continuation of Protocol #2011-17, effective until 26 October 2017; Protocol #2018-01, effective from 26 October 2017). All participants provided written informed consent.

### Data collection

Study subjects provided self-reported information regarding their age, gender, racial-ethnic background (classified as non-Hispanic White, non-Hispanic Black people, Hispanic, or other racial groups), family income (<$100,000, $100,000 and Over), season of blood collection (November 1 through April 30, or May 1 through October 31), secondhand smoke exposure, days physically active (≥5 Days/Week, at least 60 min/day), and prior medical conditions. Anthropometric measurements such as stature (in centimeters), body mass (in kilograms), and abdominal circumference (in centimeters) were obtained during the Mobile Examination Center (MEC) assessment; BMI was derived by dividing the weight in kilograms by the square of height in meters.

### Biological sample analysis

Venous blood samples were collected from participants at the NHANES MEC facility. The concentrations of 25(OH)D_2_ and 25(OH)D_3_ were determined through high-performance liquid chromatography coupled with tandem mass spectrometry (HPLC-MS/MS). Quantitative estimation was performed by comparing the response ratio (analytic peak area/internal standard peak area) of unknown samples with that of known analytic concentrations in calibration solutions (25). The total serum vitamin D [25(OH)D] level was calculated as the combined concentrations of 25(OH)D_2_ and 25(OH)D_3_. FPG, lipid profiles (HDL-C, triglycerides [TG], total cholesterol [TC], low-density lipoprotein Cholesterol [LDL-C]), liver and renal function indicators (alanine aminotransferase [ALT], gamma-glutamyl transferase [GGT], creatinine) were measured using standardized laboratory methods. Retinol levels in serum were analyzed using an adapted high-performance liquid chromatography method with photodiode array detection, complemented by spectrophotometric techniques for quantitative assessment.

### Defining MAFLD

Given the lack of explicit international consensus, hepatic steatosis was identified using a median controlled attenuation parameter (CAP) score of ≥248 dB/m, as this threshold demonstrated reliable diagnostic accuracy in a recent meta-analysis of individual patient data (26). Following the latest expert consensus (27), MAFLD in adolescents was defined by the coexistence of hepatic steatosis (assessed via CAP) along with one or more of the following conditions: overweight or obesity [BMI > 1 SD above the World Health Organization growth reference median (28) or as a waist circumference > 90th percentile for age and sex (29)], prediabetes and diabetes [(1) a previous diagnosis of diabetes; (2) a HbA1c level ≥ 5.7% (48 mmol/mol); or (3) a FPG ≥ 100 mg/dL (30)], and the presence of two or more metabolic disturbances. These metabolic disturbances encompassed increase BP (a systolic BP > 130 mmHg and/or a diastolic BP > 85 mmHg), triglyceride concentrations ≥ 150 mg/dL, HDL-cholesterol levels below 40 mg/dL, and a triglycerides-to-HDL cholesterol ratio exceeding 2.25 (with adult MAFLD criteria extended to adolescents aged 16 years and above).

### Statistical analysis

Given a complex, multi-stage sample design used in the NHANES, we applied appropriate sample weights in all analyses to account for clustering, stratification, non-response, and oversampling population. Continuous variables were expressed presented as weighted mean (standard error), categorical variables were presented as unweighted frequencies (%). Chi-squared test with Rao & Scott's second-order correction; Wilcoxon rank-sum test for complex survey samples.

Weighted restricted cubic spline regression models with three knots were used to further examine the nonlinear relationships of 25(OH)D and retinol with MAFLD. Non-linearity tested by including a quadratic term in the regression models. Survey-weight adjusted multivariable logistic regressions were performed to determine the independent associations between the 25(OH)D or retinol (both continuous variables and dichotomous variable) and MAFLD. Confounders were selected based on clinical significance and prior epidemiological evidence. Pre-specified subgroup analyses were conducted. Interaction across subgroups was tested using the likelihood ratio test. Moreover, interaction spline was produced by using the estimated odds ratio (OR) for MAFLD of a logistic model with a 25(OH)D—retinol level interaction term. Missing covariates were addressed using imputation by chained equations (31). All covariates had missing rates below 5%, except for physical activity (missing 14.5%). Sensitivity analyses were conducted through complete-case exclusion. Furthermore, an additional sensitivity analysis was performed to assess the robustness of findings to unmeasured confounding using the E-value methodology of VanderWeele and Ding (32).

A 2-tailed *p* ≤ 0.05 was considered statistically significant, and the analyses were performed using the “survey”, “rms”, and “interaction RCS” package of R software (Version 4.2.2, http://www.R-project.org, The R Foundation) and Free Statistics analysis platform (Version 2.1.1, Beijing, China, http://www.clinicalscientists.cn/freestatistics).

## Results

### Characteristics of study participants

Table 1 outlines the characteristics of the study participants (*N* = 950), categorized by 25(OH)D status. The overall mean age was 15.03 years, with a slight variation across 25(OH)D categories. The majority of participants were non-Hispanic Black people (50.60%), followed by non-Hispanic White (12.92%). Ethnic distribution significantly differed by 25(OH)D status, with Hispanic-Mexican Americans and non-Hispanic Black peoples more prevalent in the lower 25(OH)D categories. Overweight and abdominal obesity were more common in participants with lower 25(OH)D levels, while metabolic abnormalities and steatosis showed a trend toward higher prevalence in these groups. Notably, the prevalence of MAFLD was significantly higher in individuals with lower 25(OH)D levels, highlighting a potential association between vitamin D deficiency and liver health.

**Table 1:** Characteristics of study participants, divided by 25(OH)D status.

**Variables**	**Overall**	**25(OH)D**			***P* value**
		<**50 nmol/L**	**50**~**75 nmol/L**	≥**75 nmol/L**	
N, weighted	25,217,512	7,222,559	12,579,686	5,415,268	
Sex-girls, *n* (%)	416 (48.58%)	191 (55.92%)	170 (46.23%)	55 (44.26%)	0.296
Age, year	15.03 (0.07)	15.15 (0.17)	14.89 (0.09)	15.19 (0.21)	0.152
**Ethnicity, (%)**					<0.001
Hispanic-Mexican American	164 (16.91%)	74 (24.99%)	80 (17.52%)	10 (4.74%)	
Other	250 (19.57%)	107 (24.53%)	121 (21.98%)	22 (7.36%)	
Non-Hispanic Black people	264 (50.60%)	32 (18.25%)	133 (53.35%)	99 (87.36%)	
Non-Hispanic White	184 (12.92%)	134 (32.24%)	48 (7.15%)	2 (0.55%)	
Education; more than 9th grade (%)	344 (40.63%)	149 (41.88%)	138 (39.15%)	57 (42.40%)	0.806
**Family income (%)**					0.001
<$100,000	644 (68.48%)	262 (71.63%)	284 (69.92%)	98 (60.95%)	
$100,000 and over	162 (26.19%)	52 (17.44%)	82 (27.01%)	28 (35.95%)	
**Season of blood collection, (%)**					<0.001
November 1 through April 30	395 (46.92%)	186 (57.03%)	183 (51.09%)	26 (23.76%)	
May 1 through October 31	467 (53.08%)	161 (42.97%)	199 (48.91%)	107 (76.24%)	
Secondhand smoke exposure, (%)	251 (30.28%)	95 (30.59%)	108 (30.11%)	48 (30.23%)	
Physically active (≥5 Days/week, at least 60 min/day), (%)	210 (24.31%)	58 (14.37%)	103 (24.69%)	49 (36.70%)	
TG, mg/dL	73.00 (54.00, 106.00)	68.00 (50.00, 105.75)	74.46 (54.00, 107.00)	74.55 (60.00, 103.46)	0.025
HDL-C, mg/dL	52.19 (0.73)	51.24 (0.62)	51.45 (0.94)	55.25 (1.46)	0.025
TC, mg/dL	155.59 (1.60)	155.17 (1.73)	154.81 (2.52)	157.97 (1.51)	0.30
LDL-C, mg/dL	88.11 (1.24)	89.45 (1.58)	87.39 (2.16)	88.01 (1.25)	0.77
ALT, U/L	13.00 (11.00, 17.00)	13.00 (10.00, 19.02)	13.00 (11.00, 17.00)	13.00 (11.00, 15.66)	0.95
GGT, U/L	12.00 (10.00, 16.00)	13.00 (10.00, 20.00)	12.00 (10.00, 15.00)	12.00 (10.00, 14.00)	0.029
Creatinine, mg/dL	0.69 (0.59, 0.80)	0.66 (0.58, 0.77)	0.67 (0.59, 0.80)	0.72 (0.62, 0.83)	0.016
25(OH)D, nmol/L	62.01 (1.56)	37.71 (0.63)	62.46 (0.28)	93.40 (2.02)	<0.001
Retinol, ug/dL	42.95 (0.39)	39.43 (0.57)	43.30 (0.70)	46.81 (0.91)	<0.001
Overweight, (%)	395 (42.13%)	182 (53.73%)	163 (40.80%)	50 (29.69%)	0.001
Abdominal obesity, (%)	447 (50.14%)	192 (58.85%)	196 (50.92%)	59 (36.51%)	0.001
Prediabetes and diabetes, (%)	215 (23.14%)	95 (28.00%)	92 (21.55%)	28 (20.36%)	0.127
Dyslipidemia, (%)	204 (23.92%)	80 (24.77%)	95 (26.61%)	29 (16.44%)	0.112
Metabolic abnormalities, (%)	280 (34.29%)	117 (37.74%)	125 (35.65%)	38 (26.32%)	0.083
Steatosis, (%)	239 (24.78%)	114 (34.63%)	102 (23.63%)	23 (14.29%)	<0.001
MAFLD prevalence, *n* (%)	214 (21.90%)	104 (31.66%)	90 (21.08%)	20 (10.81%)	<0.001

TG, Triglycerides; HDL-C, High-Density Lipoprotein Cholesterol; TC, Total Cholesterol; LDL-C, Low-Density Lipoprotein Cholesterol; ALT, Alanine Aminotransferase; GGT, Gamma-Glutamyl Transferase; 25(OH)D, 25-Hydroxyvitamin D.

Continuous variables were expressed presented as weighted mean (standard error) or Median (IQR), categorical variables were presented as unweighted frequencies and weighted percentage (%). Chi-squared test with Rao & Scott's second-order correction; Wilcoxon rank-sum test for complex survey samples.

### Vitamin D levels and MAFLD

**Figure 2:**
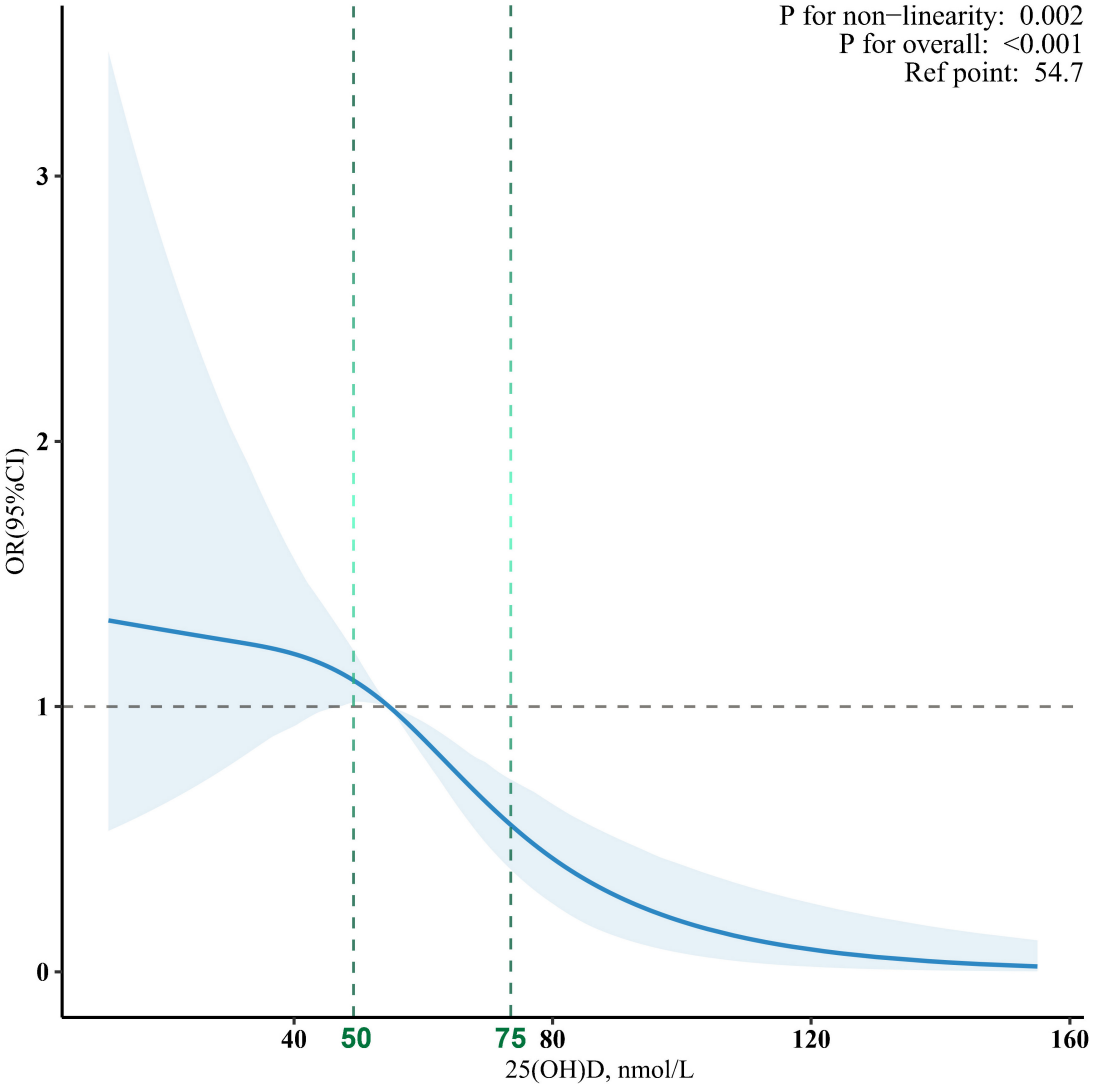
The dose-response associations between 25(OH)D levels and MAFLD. Analyses were adjusted for age, sex, ethnicity, education, season of blood collection, income, secondhand smoke, physically active, low-density lipoprotein cholesterol, alanine aminotransferase, gamma-glutamyl transferase, and creatinine.

The nonlinear curve fitting analysis established a significant association between 25(OH)D levels and MAFLD risk (Figure 2). The overall association was highly significant (*p* < 0.001), with a notable *p*-value for non-linearity at 0.002, suggesting a complex relationship rather than a simple linear trend. To further investigate this association, we conducted logistic regression analysis examining 25(OH)D levels and MAFLD prevalence in U.S. adolescents (Table 2). The analysis revealed a significant inverse dose-response relationship: each 5 nmol/L increase in 25(OH)D was associated with a 10% reduction in MAFLD risk after adjusting for potential confounders (adjusted OR = 0.90, 95% CI: 0.85~0.95, *p* < 0.001). When examining specific vitamin D thresholds, adolescents with 25(OH)D levels <50 nmol/L (reference group) exhibited the highest MAFLD risk, while those with optimal levels (≥ 75 nmol/L) showed a remarkable 57% risk reduction (OR = 0.43, 95% CI: 0.22~0.82, *p* = 0.011). The dose-response nature of this protective association was further confirmed by significant trend analysis results (*p* for trend <0.05).

**Table 2:** The associations of 25(OH)D level with MAFLD in US adolescents.

**Variable**	**Crude**	**Crude**	**Adjusted**	**Adjusted**	**Adjusted**
	**OR (95%CI)**	**P value**	**OR (95%CI)**	**P value**	**E value** ^a^
25(OH)D, per 5 nmol/L	0.91 (0.87–0.95)	<0.001	0.90 (0.85–0.95)	<0.001	1.46
**25(OH)D, nmol/L**					
<50	1(Ref)		1(Ref)		
50–75	0.72 (0.52–1)	0.051	0.80 (0.54–1.2)	0.285	-
≥75	0.41 (0.24–0.7)	0.001	0.43 (0.22–0.82)	0.011	4.08
*P* for trend		<0.001		0.017	
**25(OH)D, Quartile**					
Q1	1(Ref)		1(Ref)		
Q2	0.90 (0.60–1.36)	0.625	0.90 (0.56–1.45)	0.675	-
Q3	0.65 (0.42–0.99)	0.044	0.68 (0.41–1.13)	0.133	-
Q4	0.36 (0.22–0.57)	<0.001	0.38 (0.21–0.69)	0.002	4.70
*P* for trend		<0.001		0.001	

Odds ratio (OR), 95% confidence interval (95% CI), and p for trend values were estimated via logistic regression. Analyses were adjusted for age, sex, ethnicity, education, season of blood collection, income, secondhand smoke, physically active, low-density lipoprotein cholesterol, alanine aminotransferase, gamma-glutamyl transferase, and creatinine.

^a^The E-value was not calculated due to lack of statistical significance in the P-value.

### Subgroup analyses and interactions

**Figure 3:**
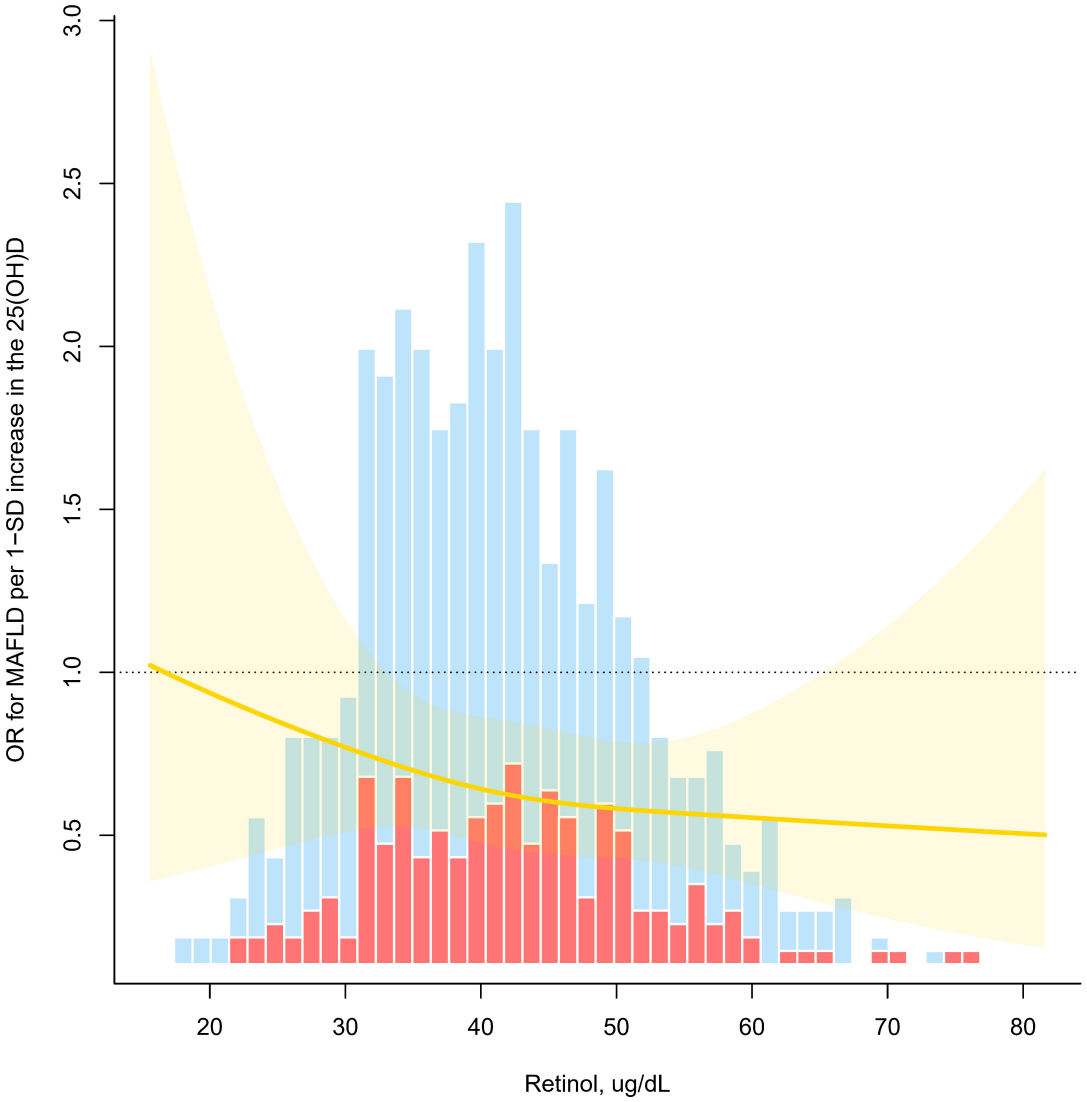
Odds ratio (OR) of vitamin D for MAFLD as a function of retinol levels. OR for MAFLD per 1-SD increase in the 25(OH)D (*P* for interaction <0.05). Estimates derived from adjusted logistic models with restricted cubic splines using knots at tertiles (33rd, 67th).

A significant retinol-dependent association between 25(OH)D levels and MAFLD was observed in interacting fitted curves, the 25(OH)D had a significantly stronger association with MAFLD at higher retinol levels (*p* for interaction <0.05, Figure 3). Specifically, in stratified analysis, children have a higher retinol nutritional status exhibited an inverse relationship between high 25(OH)D levels and MAFLD (OR = 0.22), contrasting with no significant effect for low retinol levels groups (OR = 0.65) (Table 3). Besides, age group, sex, ethnicity, overweight, abdominal obesity, season of blood collection, and physically active did not influence the relationship (p for interaction > 0.05).

**Table 3:** Associations of 25(OH)D with MAFLD, divided by age, sex and retinol status.

**Subgroup**	OR (95%CI)^*^	***P* value**	***P* for interaction**
**Age**			0.402
<15 y	0.61 (0.25–1.47)	0.271	
≥15 y	0.41 (0.18–0.93)	0.032	
**Sex**			0.978
Boys	0.55 (0.25–1.19)	0.13	
Girls	0.47 (0.19–1.16)	0.103	
**Ethnicity**			0.546
Non-Hispanic Black people	0.38 (0.17–0.84)	0.017	
All Other Ethnicity	0.58 (0.23–1.49)	0.256	
**Retinol status**			0.047
Low (<Quartile 4)	0.65 (0.32–1.29)	0.214	
High (≥Quartile 4)	0.22 (0.07–0.7)	0.011	
**Overweight**			0.296
No	2.06 (0.41–10.34)	0.381	
Yes	0.43 (0.21–0.87)	0.019	
**Abdominal obesity**			0.091
No	6.58 (0.87–49.91)	0.068	
Yes	0.47 (0.24–0.92)	0.028	
**Season of blood collection**			0.257
November 1 through April 30	0.23 (0.05–0.96)	0.045	
May 1 through October 31	0.62 (0.31–1.25)	0.179	
**Physically active**			0.101
<5 days/week, at least 60 min/day	0.36 (0.17–0.76)	0.008	
≥5 days/week, at least 60 min/day	0.86 (0.29–2.54)	0.787	

^*^OR was calculated by independent variable 25(OH)D ≥ 75 nmol/L vs. 25(OH)D <75 nmol/L.

Odds ratio (OR), 95% confidence interval (95% CI), and p for interaction were estimated via logistic regression. Analyses were adjusted for age, sex, ethnicity, education, season of blood collection, income, secondhand smoke, physically active, low-density lipoprotein cholesterol, alanine aminotransferase, gamma-glutamyl transferase, and creatinine.

### Retinol levels and MAFLD

In light of the combined health effects of vitamin A and vitamin D nutritional status in children, this study further analyzed the relationship between retinol levels and MAFLD. However, the results did not reach statistical significance. In the restricted cubic spline analysis, the overall *p* = 0.608 (Supplementary Figure 1). In the logistic regression analysis (Supplementary Table 1), the adjusted OR for retinol per 5 μg/dL increase was 1.03 (95% CI: 0.93-1.14, *p* = 0.562). Quartile analysis revealed similar trends, with Q1 as the reference group; Q2 (OR = 0.90, 95% CI: 0.40–2.03, *p* = 0.746), Q3 (OR = 1.25, 95% CI: 0.52–3.03, *p* = 0.517), and Q4 (OR = 1.09, 95% CI: 0.49–2.43, *p* = 0.790) showed no significant differences. The trend analysis supported these findings (*p* for trend > 0.05).

## Discussion

This study, based on NHANES 2017–2018 data, comprehensively evaluated the association between serum 25(OH)D levels and MAFLD risk in adolescents. The results revealed an inverse association between serum 25(OH)D levels and MAFLD risk, demonstrating a non-linear dose-response relationship that was modified by factors such as retinol levels. The results of this study will provide new scientific evidence for the potential role of vitamin D in the onset and progression of MAFLD.

Our study found significant differences in serum 25(OH)D levels across ethnic and socioeconomic groups. Higher concentrations were most common in Non-Hispanic Black people individuals, those with lower family incomes, and people who were not overweight or abdominally obese. Notably, we observed a significantly higher prevalence of MAFLD in populations with lower serum 25(OH)D levels, with MAFLD prevalence progressively decreasing as 25(OH)D levels increased. Similar findings have been reported in previous studies; a prospective study demonstrated that lower 25(OH)D concentrations were significantly associated with NAFLD in adolescents, and this association was independent of obesity (33). Additionally, an observational study conducted in Brazil found a high prevalence of vitamin D deficiency and insufficiency among obese adolescents, which was closely associated with metabolic changes (34).Vitamin D may exert a protective effect against hepatic fat deposition by improving insulin sensitivity (35), inhibiting inflammatory responses, and regulating lipid metabolism (36). Therefore, maintaining adequate vitamin D status may be an important metabolic regulatory factor for reducing the risk of MAFLD in adolescents. Additionally, race and family income have been shown to be closely related to the risk of MAFLD (37, 38). Thus, these factors were adjusted for as covariates in our study. The results demonstrated a non-linear dose-response relationship between serum 25(OH)D levels and MAFLD risk in adolescents, which is consistent with previous research. A prospective cohort study in China also found a non-linear negative association between serum 25(OH)D concentrations and all-cause mortality in patients with MAFLD or NAFLD (39). Our findings further demonstrate that vitamin D deficiency exerts a more pronounced adverse effect on MAFLD risk, whereas its protective efficacy appears to stabilize beyond a specific 25(OH)D concentration threshold. This suggests a potential “threshold effect” of vitamin D in regulating metabolic homeostasis. Adequate levels are essential for protection, but higher concentrations may not provide extra benefits. These findings highlight the need for targeted vitamin D interventions. It is more effective to focus on deficient groups rather than supplementing those who already have adequate levels.

This study explored the moderating effects of age levels on the relationship between vitamin D and MAFLD. Age-stratified analysis showed that the protective effect of vitamin D was more significant in adolescents aged ≥ 15 years, while no statistically significant association was observed in the group <15 years old. This age-dependent differential effect may be attributed to pubertal development changes and hormonal fluctuations (40). The adolescent period represents a critical window of metabolic transformation, characterized by substantial increases in growth hormone and sex hormone levels, coupled with progressive insulin resistance (41). These physiological changes potentially enhance susceptibility to MAFLD, thereby increasing hepatic fat accumulation risk (42). Indeed, puberty has been identified as a high-risk period for the development and progression of obesity and metabolic dysregulation (43). Vitamin D acts as a metabolic modulator and may enhance protection by regulating insulin sensitivity, inflammation, and lipid metabolism (44, 45).

Interestingly, we further found differential associations between vitamin D and MAFLD across retinol concentration subgroups. Specifically, among individuals with higher retinol levels, the protective effect of vitamin D against MAFLD was significantly enhanced, while no similar effect was observed in those with lower retinol levels. This observation implies that retinol may enhance vitamin D metabolic regulatory capacity through synergistic interactions, consequently mitigating MAFLD risk. As the main active form of vitamin A, retinol is an essential micronutrient for mammals, primarily stored in the liver, kidneys, and pulmonary tissues (46). Its physiological significance extends to the regulation of cellular differentiation, immune modulation, and lipid homeostasis (47). Studies have confirmed that retinol plays an important role in regulating liver lipid metabolism and inflammatory response (48, 49). A Dutch study (49) demonstrated that retinol ameliorates lipid dysmetabolism and inflammation through modulation of hepatic signaling pathways, providing mechanistic insights into its potential metabolic protective properties. Although retinol can enhance the protective effect of vitamin D, this study did not find a significant independent association between retinol and MAFLD risk. This suggests that retinol may not directly influence MAFLD pathogenesis but rather exerts its metabolic protective effects indirectly through vitamin D pathway modulation. This interpretation aligns with Zeng et al.'s finding (50), which indicates that retinol enhances vitamin D's effects mainly by boosting vitamin D receptor activity, rather than through direct involvement in MAFLD pathways. In summary, retinol may play a synergistic role in vitamin D-mediated metabolic homeostasis regulation by enhancing the activity of the vitamin D signaling pathway, but its preventive effect on MAFLD alone may be limited. Therefore, future nutritional intervention strategies should pay more attention to the interaction between fat-soluble vitamins, especially the synergistic supplementation of vitamin A and vitamin D, in order to more effectively reduce the risk of MAFLD in adolescents.

Based on the nationally representative data from NHANES, this study systematically evaluated the relationship between vitamin D levels and the risk of MAFLD in adolescents and further explored the potential interactions of age and retinol. The results highlighted the potential protective role of vitamin D in the risk of MAFLD in individuals with high retinol levels, where the metabolic protective effects were more significant. However, this study still has some limitations. First, the cross-sectional nature of this study fundamentally limits causal inference, as reverse causality cannot be ruled out. Consequently, caution is warranted when interpreting these findings. Therefore, future prospective cohort studies or intervention studies should be conducted to further clarify the causal role of vitamin D in the occurrence and development of MAFLD and the related biological mechanisms. Second, this study did not investigate factors such as dietary vitamin D intake, sunlight exposure, socioeconomic indicators, and lifestyle factors. Although multiple confounding variables were adjusted for in the statistical models, certain potential confounders remained incompletely controlled. However, we performed sensitivity analyses using the E-value methodology to assess the robustness of our findings to unmeasured confounding. These analyses indicated that our results are less likely to be reversed by unadjusted confounding factors. Third, the ethnic distribution of the sample limits the generalizability of our findings. Specifically, non-Hispanic Black peoples represented 50.6% of participants and Hispanic Mexican Americans 23.1%, while Asian and other ethnic groups were underrepresented. As vitamin D metabolism varies with skin pigmentation and cultural practices, these results should be generalized to other populations with caution. Finally, it should be noted that the observed associations of 25(OH)D with MAFLD, divided by retinol status, may be subject to false positives attributable to multiple testing. In light of this, we strongly recommend that future research prioritize the use of prospective validation cohorts in conjunction with mechanistic studies. This approach will enable a more robust exploration of the association between 25(OH)D and MAFLD.

In conclusion, our investigation revealed a significant inverse correlation between 25(OH)D concentrations and MAFLD risk in adolescent populations, characterized by a nonlinear dose-response relationship. Notably, a trend of enhanced protective effects of vitamin D was observed in the subgroup with higher retinol levels. This exploratory finding suggests that retinol may potentiate the biological activity of vitamin D through synergistic mechanisms, providing a preliminary hypothesis for future research on fat-soluble vitamin interactions. However, these observations require further validation through prospective studies combined with mechanistic experiments. Our results indicate that maintaining adequate vitamin D levels may have positive implications for MAFLD prevention in adolescents. While focusing on specific nutrient supplementation, future research could further explore the potential value of synergistic effects among multiple micronutrients. Such comprehensive nutritional intervention approaches may offer more comprehensive strategic references for the prevention and management of metabolic diseases in adolescents.

## Data Availability

The original contributions presented in the study are included in the article/Supplementary material, further inquiries can be directed to the corresponding authors.

## References

[1] Younossi ZM Golabi P Paik JM Henry A Van Dongen C Henry L. The global epidemiology of nonalcoholic fatty liver disease (Nafld) and nonalcoholic steatohepatitis (Nash): a systematic review. Hepatology. (2023) 77:1335–47. 10.1097/HEP.0000000000000004 36626630 PMC10026948

[2] Eslam M Newsome PN Sarin SK Anstee QM Targher G Romero-Gomez M. A new definition for metabolic dysfunction-associated fatty liver disease: an international expert consensus statement. J Hepatol. (2020) 73:202–9. 10.1016/j.jhep.2020.07.045 32278004

[3] Engin A. The definition and prevalence of obesity and metabolic syndrome. Adv Exp Med Biol. (2017) 960:1–17. 10.1007/978-3-319-48382-5_1 28585193

[4] Ge X Zheng L Wang M Du Y Jiang J. Prevalence trends in non-alcoholic fatty liver disease at the global, regional and national levels, 1990-2017: a population-based observational study. BMJ Open. (2020) 10:e036663. 10.1136/bmjopen-2019-036663 32747349 PMC7402189

[5] Wang MF Wan B Wu YL Huang JF Zhu YY Li YB. Clinic-pathological features of metabolic associated fatty liver disease with hepatitis B virus infection. World J Gastroenterol. (2021) 27:336–44. 10.3748/wjg.v27.i4.336 33584066 PMC7852586

[6] Yang K Song M. New Insights into the pathogenesis of metabolic-associated fatty liver disease (Mafld): gut-liver-heart crosstalk. Nutrients. (2023) 15:3970. 10.3390/nu15183970 37764755 PMC10534946

[7] Eliades M Spyrou E. Vitamin D: a new player in non-alcoholic fatty liver disease? World J Gastroenterol. (2015) 21:1718–27. 10.3748/wjg.v21.i6.1718 25684936 PMC4323447

[8] Turner AG Anderson PH Morris HA. Vitamin D and bone health. Scand J Clin Lab Invest Suppl. (2012) 243:65–72. 22536765 10.3109/00365513.2012.681963

[9] Ebeling PR. Vitamin D and bone health: epidemiologic studies. Bonekey Rep. (2014) 3:511. 10.1038/bonekey.2014.6 24818003 PMC4015454

[10] Kwok RM Torres DM Harrison SA. Vitamin D and nonalcoholic fatty liver disease (Nafld): is it more than just an association? Hepatology. (2013) 58:1166–74. 10.1002/hep.26390 23504808

[11] Szymczak-Pajor I Drzewoski J Sliwinska A. The molecular mechanisms by which vitamin D prevents insulin resistance and associated disorders. Int J Mol Sci. (2020) 21:6644. 10.3390/ijms21186644 32932777 PMC7554927

[12] Jones G Prosser DE Kaufmann M. The activating enzymes of vitamin D metabolism (25-and 1α-Hydroxylases). In: Vitamin D. London: Elsevier (2018). p. 57–79.

[13] Holick MF. Vitamin D deficiency. N Engl J Med. (2007) 357:266–81. 10.1056/NEJMra070553 17634462

[14] Trimarco V Manzi MV Mancusi C Strisciuglio T Fucile I Fiordelisi A. Insulin resistance and vitamin D deficiency: a link beyond the appearances. Front Cardiovasc Med. (2022) 9:859793. 10.3389/fcvm.2022.859793 35369303 PMC8968037

[15] Mozos I Marginean O. Links between vitamin D deficiency and cardiovascular diseases. Biomed Res Int. (2015) 2015:109275. 10.1155/2015/109275 26000280 PMC4427096

[16] Al-Sumaih I Johnston B Donnelly M O'Neill C. The relationship between obesity, diabetes, hypertension and vitamin D deficiency among saudi arabians aged 15 and over: results from the saudi health interview survey. BMC Endocr Disord. (2020) 20:81. 10.1186/s12902-020-00562-z 32503594 PMC7275458

[17] Gradillas-García A Álvarez J Rubio JA de Abajo FJ. Relationship between vitamin D deficiency and metabolic syndrome in adult population of the community of madrid. Endocrinologí*a y Nutrición*. (2015) 62:180–7. 10.1016/j.endoen.2015.04.004 25726369

[18] Chung GE Kim D Kwak MS Yang JI Yim JY Lim SH. The serum vitamin D level is inversely correlated with nonalcoholic fatty liver disease. Clin Mol Hepatol. (2016) 22:146–51. 10.3350/cmh.2016.22.1.146 27044765 PMC4825160

[19] Ciardullo S Muraca E Cannistraci R Perra S Lattuada G Perseghin G. Low 25 (Oh) vitamin D levels are associated with increased prevalence of nonalcoholic fatty liver disease and significant liver fibrosis. Diabetes Metab Res Rev. (2023) 39:e3628. 10.1002/dmrr.3628 36815587

[20] Cho YH Kim JW Shim JO Yang HR Chang JY Moon JS. Association between vitamin D deficiency and suspected nonalcoholic fatty liver disease in an adolescent population. Pediatr Gastroenterol Hepatol Nutr. (2019) 22:233–41. 10.5223/pghn.2019.22.3.233 31110956 PMC6506433

[21] Gutiérrez-Cuevas J Santos A Armendariz-Borunda J. Pathophysiological molecular mechanisms of obesity: a link between Mafld and Nash with cardiovascular diseases. Int J Mol Sci. (2021) 22:11629. 10.3390/ijms222111629 34769060 PMC8583943

[22] Li H Xiao P Cheng H Zhao X Yan Y Liu J. Central body fat deposits are associated with poor vitamin D status in Chinese children and adolescents. Nutrition. (2022) 99–100:111651. 10.1016/j.nut.2022.111651 35588652

[23] Gofton C Upendran Y Zheng MH George J. Mafld: how is it different from Nafld? Clin Mol Hepatol. (2023) 29:S17–S31. 10.3350/cmh.2022.0367 36443926 PMC10029949

[24] National Center for Health Statistics. National Health and Nutrition Examination Survey. (2023). Available online at: http://www.cdc.gov/nchs/nhanes/ (Accessed September 1, 2023).

[25] CDC. Laboratory Procedure Manual. (2023). Available online at: https://wwwn.cdc.gov/nchs/data/nhanes/public/2017/labmethods/VID-J-MET-508.pdf (Accessed September 1, 2023).

[26] Karlas T Petroff D Sasso M Fan JG Mi YQ de Lédinghen V. Individual patient data meta-analysis of controlled attenuation parameter (Cap) technology for assessing steatosis. J Hepatol. (2017) 66:1022–30. 10.1016/j.jhep.2016.12.022 28039099

[27] Eslam M Alkhouri N Vajro P Baumann U Weiss R Socha P. Defining paediatric metabolic (dysfunction)-associated fatty liver disease: an international expert consensus statement. Lancet Gastroenterol Hepatol. (2021) 6:864–73. 10.1016/S2468-1253(21)00183-7 34364544

[28] World Health Organization Bmi-for-Age Charts (5-19years) (2022). Available online at: https://www.who.int/tools/growth-reference-data-for-5to19-years/indicators/bmi-for-age (Accessed February 21, 2022).

[29] Xi B Zong X Kelishadi R Litwin M Hong YM Poh BK. International waist circumference percentile cutoffs for central obesity in children and adolescents aged 6 to 18 years. J Clin Endocrinol Metab. (2020) 105:e1569–83. 10.1210/clinem/dgaa578 31723976 PMC7059990

[30] American Diabetes Association Professional Practice Committee. 2. classification and diagnosis of diabetes: standards of medical care in diabetes-2022. Diabetes Care (2022) 45:S17–s38. 10.2337/dc22-S002 34964875

[31] van Buuren S Groothuis-Oudshoorn K. Mice: multivariate imputation by chained equations in R. J Statist Softw. (2011) 45:1–67. 10.18637/jss.v045.i03

[32] VanderWeele TJ Ding P. Sensitivity analysis in observational research: introducing the E-value. Ann Intern Med. (2017) 167:268–74. 10.7326/M16-2607 28693043

[33] Black LJ Jacoby P She Ping-Delfos WC Mori TA Beilin LJ Olynyk JK. Low serum 25-hydroxyvitamin D concentrations associate with non-alcoholic fatty liver disease in adolescents independent of adiposity. J Gastroenterol Hepatol. (2014) 29:1215–22. 10.1111/jgh.12541 24611991

[34] Teixeira JS Bull Ferreira Campos A Cordeiro A Pereira SE Saboya CJ Ramalho A. Vitamin D nutritional status and its relationship with metabolic changes in adolescents and adults with severe obesity. Nutr Hosp. (2018) 35:847–53. 10.20960/nh.1657 30070873

[35] Barchetta I Cimini FA Cavallo MG. Vitamin D and metabolic dysfunction-associated fatty liver disease (Mafld): an update. Nutrients. (2020) 12:3302. 10.3390/nu12113302 33126575 PMC7693133

[36] Bozic M Guzman C Benet M Sanchez-Campos S Garcia-Monzon C Gari E. Hepatocyte vitamin D receptor regulates lipid metabolism and mediates experimental diet-induced steatosis. J Hepatol. (2016) 65:748–57. 10.1016/j.jhep.2016.05.031 27245430

[37] Shaheen M Schrode KM Pan D Kermah D Puri V Zarrinpar A. Sex-specific differences in the association between race/ethnicity and Nafld among us population. Front Med. (2021) 8:795421. 10.3389/fmed.2021.795421 34926533 PMC8674562

[38] Lee EY Nguyen VH Cheung R Nguyen MH. Trends of chronic liver diseases by income level and socioeconomic factors in the united states: a population-based study. Aliment Pharmacol Ther. (2024) 60:1374–87. 10.1111/apt.18242 39238267

[39] Zhang JJ Yu HC Li Y Zhang YB Geng TT Lu Q. Association between Serum 25-hydroxy vitamin D concentrations and mortality among individuals with metabolic dysfunction-associated fatty liver disease: a prospective cohort study. Am J Clin Nutr. (2022) 116:1409–17. 10.1093/ajcn/nqac260 36107812

[40] Pires LV Gonzalez-Gil EM Anguita-Ruiz A Bueno G Gil-Campos M Vazquez-Cobela R. The vitamin D decrease in children with obesity is associated with the development of insulin resistance during puberty: the pubmep study. Nutrients. (2021) 13:4488. 10.3390/nu13124488 34960039 PMC8709093

[41] Scapaticci S D'Adamo E Mohn A Chiarelli F Giannini C. Non-alcoholic fatty liver disease in obese youth with insulin resistance and type 2 diabetes. Front Endocrinol. (2021) 12:639548. 10.3389/fendo.2021.639548 33889132 PMC8056131

[42] Sakurai Y Kubota N Yamauchi T Kadowaki T. Role of insulin resistance in Mafld. Int J Mol Sci. (2021) 22:4156. 10.3390/ijms22084156 33923817 PMC8072900

[43] Cui X Sun X Li Q Chen Z. Changes in blood glucose and lipid metabolism levels in children with central precocious puberty and its correlation with obesity. Front Pediatr. (2024) 12:1488522. 10.3389/fped.2024.1488522 39840310 PMC11747382

[44] Argano C Mirarchi L Amodeo S Orlando V Torres A Corrao S. The role of vitamin D and its molecular bases in insulin resistance, diabetes, metabolic syndrome, and cardiovascular disease: state of the art. Int J Mol Sci. (2023) 24:15485. 10.3390/ijms242015485 37895163 PMC10607188

[45] Namakin K Hosseini M Zardast M Mohammadifard M. Vitamin D effect on ultrasonography and laboratory indices and biochemical indicators in the blood: an interventional study on 12 to 18-year-old children with fatty liver. Pediatr Gastroenterol Hepatol Nutr. (2021) 24:187–96. 10.5223/pghn.2021.24.2.187 33833974 PMC8007841

[46] Murat G Sule A. Understanding the role of vitamin a and its precursors in the immune system. Nutr Clinique et Métabolisme. (2022) 36:89–98. 10.1016/j.nupar.2021.10.002

[47] Chen W Chen G. The roles of vitamin a in the regulation of carbohydrate, lipid, and protein metabolism. J Clin Med. (2014) 3:453–79. 10.3390/jcm3020453 26237385 PMC4449691

[48] Petiz LL Kunzler A Bortolin RC Gasparotto J Matté C Moreira JCF. Role of vitamin A oral supplementation on oxidative stress and inflammatory response in the liver of trained rats. Appl Physiol Nutr Metab. (2017) 42:1192–200. 10.1139/apnm-2017-0193 28742973

[49] Saeed A Dullaart RPF Schreuder T Blokzijl H Faber KN. Disturbed vitamin A metabolism in non-alcoholic fatty liver disease (Nafld). Nutrients. (2017) 10:29. 10.3390/nu10010029 29286303 PMC5793257

[50] Zeng Y Luo M Pan L Chen Y Guo S Luo D. Vitamin D signaling maintains intestinal innate immunity and gut microbiota: potential intervention for metabolic syndrome and Nafld. Am J Physiol Gastrointest Liver Physiol. (2020) 318:G542–G53. 10.1152/ajpgi.00286.2019 31984787 PMC7099486

