# Supplementary material for: Association of vitamin D levels with metabolic dysfunction-associated fatty liver disease in children aged 12–18 years
Source: Front Nutr. 2025 Nov 4;12:1615851. doi: 10.3389/fnut.2025.1615851 (PMC12624440; doi:10.3389/fnut.2025.1615851)
Supplement: Supplementary file 1 [file Supplementary_file_1.docx]

**Supplemental Materials**

**Contents**

**Supplementary Figure 1.** The dose-response associations between Retinol levels and MAFLD

**Supplementary Table 1.** The associations of retinol level with MAFLD in US adolescents


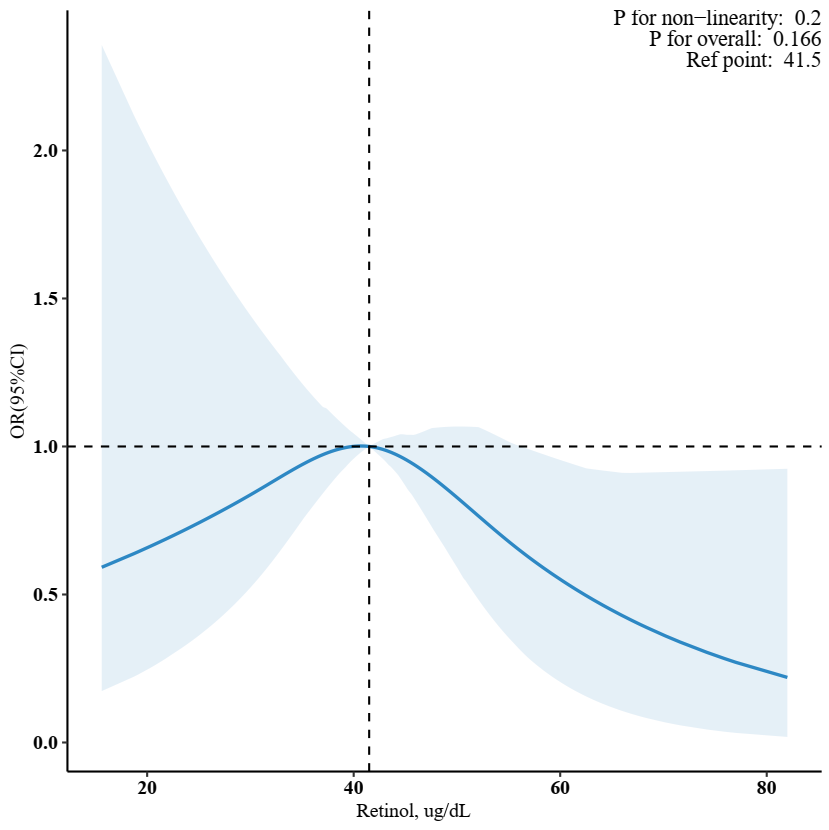


**Supplementary Figure 1. The dose-response associations between Retinol levels and MAFLD**

Analyses were adjusted for age, sex, ethnicity, education, six month time period, and income.

**Supplementary Table 1. The associations of retinol level with MAFLD in US adolescents**

| **Variable** | **Crude** |  | **Adjusted** |  |  |
| --- | --- | --- | --- | --- | --- |
|  | ***OR (95%CI)*** | ***P value*** | ***OR (95%CI)*** | ***P value*** | ***E value*** ^a^ |
| Retinol, per 5 ug/dL | 1.05 (0.98~1.14) | 0.181 | 0.96 (0.87~1.06) | 0.386 | - |
| Retinol, Quartile |  |  |  |  |  |
| Q1 | 1(Ref) |  | 1(Ref) |  |  |
| Q2 | 0.89 (0.57~1.41) | 0.632 | 0.88 (0.53~1.46) | 0.627 | - |
| Q3 | 1.26 (0.81~1.94) | 0.307 | 1.11 (0.67~1.85) | 0.683 | - |
| Q4 | 1.24 (0.80~1.92) | 0.331 | 0.76 (0.43~1.32) | 0.322 | - |
| *P* for trend |  | 0.160 |  | 0.504 |  |

Odds ratio (OR), 95% confidence interval (95% CI), and *P* for trend values were estimated via logistic regression. Analyses were adjusted for age, sex, ethnicity, education, season of blood collection, income, secondhand smoke, physically active, low-density lipoprotein cholesterol, alanine aminotransferase, gamma-glutamyl transferase, and creatinine.

^a^ The E-value was not calculated due to lack of statistical significance in the *P*-value.
